# Representation of persons experiencing homelessness and coding of homelessness in general practices: descriptive evaluation using healthcare utilisation data

**DOI:** 10.3399/BJGPO.2021.0050

**Published:** 2021-06-16

**Authors:** Rishika Kaushal, Parbir Jagpal, Saval Khanal, Neha Vohra, Richard Lowrie, Jaspal Johal, Duncan Jenkins, Karen Saunders, Vibhu Paudyal

**Affiliations:** 1 College of Medical and Dental Sciences, University of Birmingham, Birmingham, UK; 2 Division of Health Sciences, Warwick Medical School, University of Warwick, Coventry, UK; 3 NHS Greater Glasgow and Clyde, Glasgow, UK; 4 Dudley Integrated Health and Care NHS Trust, Dudley, UK; 5 Public Health England West Midlands, Birmingham, UK

**Keywords:** homeless persons, homelessness, primary health care, England, general practice

## Abstract

**Background:**

Epidemiological studies focused on primary healthcare needs of persons experiencing homelessness (PEH) are often based on data from specialist homeless healthcare services.

**Aim:**

To explore the presentation of PEH, coding of homelessness, and associated health conditions in mainstream primary care general practices in England.

**Design & setting:**

EMIS electronic database search of medical records was conducted across 48 general practices in a clinical commissioning group (CCG), representing one of the most socioeconomically deprived regions in England, which also lacks a specialist primary healthcare service for PEH.

**Method:**

Key terms and codes were used to identify PEH, their respective diagnoses across 22 health conditions, and prescribed medications over the past 4 years.

**Results:**

From a population of approximately 321 000, 43 (0.013%) people were coded as PEH, compared with a homelessness prevalence of 0.5% in the English general population. Mental health conditions were the most prevalent diagnoses among the PEH registrants (56.6%); the recorded prevalence of other common long-term conditions in PEH was lower than the levels observed in PEH registered with specialist homelessness health services.

**Conclusion:**

In a population with approximately four times higher rate of statutory homelessness, PEH representation in mainstream general practices was under-represented by several folds. As homelessness overlaps with mental health, substance misuse, and long-term health conditions, consistent coding of homelessness in medical records is imperative in order to offer tailored support and prevention actions when patients present for services.

## How this fits in

Previous qualitative studies have demonstrated PEH face multiple barriers to accessing mainstream general practices. Prevalence and characteristics of PEH registered in mainstream primary health care have not been investigated to date. There is a need to code homelessness accurately in primary care medical records to offer tailored support.

## Introduction

Homelessness can be defined as a situation where an individual does not have a secure or safe place of residence. It includes those staying in homeless shelters or those who are sleeping on the streets (‘rough sleepers’). It also includes staying in temporary accommodation such as bed and breakfasts, hostels, squats, or those ‘sofa-surfing’ between family and friends’ houses.^1^ Nearly 0.5% (280 000 people) in England are known to experience homelessness.^2^ Additionally, before the COVID-19 pandemic, approximately 5000 people slept rough in any one night in England.^3^ Poverty, substance misuse, severe mental health problems, relationship breakdowns, and childhood trauma have been shown to be both causes and consequences of homelessness.^4^

PEH are high users of emergency departments.^5^ As demonstrated by previous research, key barriers to the utilisation of primary healthcare services by PEH include: the lack of tailored services to meet their multiple complex needs; services located within buildings and based on appointment systems that are difficult for PEH to navigate; difficulties in registering with a general practice owing to lack of address; and perceived stigma and discrimination towards PEH in primary care.^6^

During the past decade, to help increase the accessibility of primary health care to PEH, specialist primary care services for individuals experiencing homelessness have been established across the UK. Such services include specialist health centres, general practices within homeless services, and mobile homeless health teams.^7,8^ Such services can include nurses, GPs, pharmacists, drug and alcohol dependence specialists, opticians, and psychologists. Some are based at fixed locations but others provide mobile clinics offering their services to hostels and day centres, thus making health care even more accessible to these individuals. However, such centres are only located in key urban areas,^7^ operate limited hours, and may not able to serve all PEH.

Previous studies based in the UK have attempted to investigate healthcare issues of PEH who present at the specialist homelessness healthcare centres.^9–11^ At present, there is a lack of research regarding the utilisation of mainstream (that is, those not specifically established for PEH) primary care general practices by PEH. In addition, little is known about the coding of homelessness when PEH present to the practices. The aim of this study was to determine the prevalence of homelessness as coded in the medical records of registrants in a cluster of general practices within a socioeconomically deprived region of England. Specific objectives were to describe the demographic characteristics, recorded prevalence of health conditions, and their commonly prescribed medicines.

## Method

This study used routinely collected data from all 48 general practices in England from within a region that represents the top decile of socioeconomically deprived areas in England. The life expectancy in the region is reported to be at least 10 years lower for males and 6 years lower for females relative to England, and has a significantly higher rate of statutory homelessness. The 48 general practices accounted for approximately 321 000 registered individuals. The statutory homeless rate in the region is reported to be four times higher than the rate of England.

Data on PEH were obtained by searching general practice-level computerised patient records (EMIS database) from the period 1 June 2015–31 May 2019 using the terminologies presented in Table 1. The terminologies were derived through discussion with clinical staff in the study practices, published literature including policy documents, and authors’ experiences of research and clinical practice with PEH. The 4 years of data collection period was selected to capture patients who are often temporarily registered in practice given the transient nature of PEH populations in primary care.^1,12,13^

**Table 1.** A list of codes used to search EMIS software to identify persons experiencing homelessness

Homeless	Sleeping in night shelter
Homelessness	Living in temporary council accommodation
No fixed abode	Living in B&B accommodation
Sleeping rough	Living in B&B accommodation
Rough sleeper	Living in lodgings
Squatter	Living in a bedsit
Lives in lodgings	Living in bedsitter

B&B = bed and breakfast.

Demographic data regarding age, sex, smoking status, and ethnic group were obtained. Medications (both acute and repeat) prescribed over the study period were also extracted from the patient medical records. The prevalence of 22 common health conditions were extracted, including cardiovascular, endocrine, respiratory, mental health, neurological, gastrointestinal disorders, infections, other diseases including rheumatoid arthritis, leg ulcers, and learning disability. Data were extracted by clinical practice staff with routine access to patient medical records and anonymised, and small numbers suppressed before electronic transfer to the research team. Data were descriptively analysed (owing to the small sample size) to identify the prevalence of PEH within the study area, their demographic characteristics, disease prevalence, and frequently prescribed medications. Data on disease prevalence were compared with UK and international homeless and the general populations from the published literature. Data on prescribed medicines were categorised as per the *British National Formulary* (BNF) chapters.^14^

## Results

Of the 320 932 patients registered in the 48 general practices, 43 registrants were coded as PEH during the 4-year study period. The majority of the PEH registrants were male (*n* = 27, 62.8%) and the mean age was 42.1 (SD 17.4) years (range 19–90 years). The majority of registrants were between the ages of 20 and 39 years. A total of 20 (46.5%) were from a White British ethnic group, followed by approximately a third from a Mixed British ethnic group (*n* = 12, 27.9%) (Table 2). A total of 69.8% currently smoke, and 70% of these were male. The majority of those who smoke were between the ages of 20 years and 39 years (*n* = 30, 64.3%).

**Table 2.** Demographic characteristics of registrants experiencing homelessness

	**Female*n* =16*n* (%**)	**Male*n* =27*n* (%**)	**All*n* =43*n* (%**)
Mean age (SD), years	42.6 (19.1)	41.7 (16.8)	42.1 (17.4)
Age range, years			
20–29	5 (31.3)	9 (33.3)	14 (32.6)
30–39	3 (18.8)	7 (25.9)	10 (23.3)
40–49	3 (18.8)	2 (7.4)	5 (11.6)
50–59	2 (12.5)	5 (18.5)	7 (16.3)
60–69	2 (12.5)	3 (11.1)	5 (11.6)
70–79	–	–	–
80–89	–	1 (3.7)	1 (2.3)
90–99	1 (6.3)	–	1 (2.3)
Ethnic group			
White British	11 (68.8)	9 (33.3)	20 (46.5)
Mixed	1 (6.3)	1 (3.7)	2 (4.7)
Mixed British	3 (18.8)	9 (33.3)	12 (27.9)
Other	1 (6.3)	2 (7.4)	3 (7.0)
Unknown	–	6 (22.2)	6 (14.0)

Mental health conditions were the most prevalent of the 53 total diagnoses (*n* = 30, 56.6%) followed by cardiovascular diseases (*n* = 7, 13.2%). Depression and alcohol dependence were the most prevalent mental health disorders (Table 3). Out of the 14 registrants who were diagnosed with a mental health disorder, such as depression, mania, hypomania, psychosis, and bipolar disorders, seven (50.0%) of these were concurrently diagnosed with substance misuse related either to alcohol, opioid, or heroin dependence.

**Table 3.** The prevalence of health conditions among the registrants

**Health conditions category, *n* (%**)	**Diagnoses**	**Prevalence,** ***n* (%)^a^**	**Prevalence from UK** **l** **iterature, %**	**Prevalence from international literature, %**
Mental health conditions,30 (56.6)	Depression	10 (18.9)	29.7, Leicester^36^11.6, Birmingham^9^50.0, Dublin^37^42.1, Glasgow^10^55.0, Edinburgh^11^33.0, Glasgow^11^	Not available
	Mental health register	–	6.5, Birmingham^9^	Not available
	Alcohol dependence	10 (18.9)	29.0, Leicester^36^21.3, Birmingham^9^53.0, Dublin^37^56.4, Glasgow^10^37.0, Edinburgh^11^54.0, Glasgow^11^	37.9, Western countries^38^
	Substance dependence	4 (7.5)	66.0, Leicester^36^13.5, Birmingham^9^33.0, Dublin^14^62.4, Glasgow^10^73.0, Edinburgh^11^62.0, Glasgow^10^	24.2, Western countries^38^
Neurological disorders,3 (5.7)				
	Epilepsy	–	1.4, Birmingham^9^8.0, Dublin^37^	8.1, Paris^39^6.0, Canada^40^
	Migraine	2 (3.8)	1.1, Birmingham^9^	25–36, Canada^41,42^
Cardiovascular disease,7 (13.2)	Hypertension	4 (7.5)	4.2, Birmingham^9^	27.0, US^43^
	Coronary heart disease	–	1.5, Birmingham^9^	Not available
	Stroke or transient ischaemic attack	1 (1.9)	0.3, Birmingham^9^	20.0, US^44^
	Heart failure	–	Not available	Not available
	Atrial fibrillation	1 (1.9)	0.2, Birmingham^9^	Not available
	Angina	–	Not available	Not available
Infections,3 (5.7)	Hepatitis C	2 (3.8)	11.3, Leicester^36^6.3, Birmingham^9^23.0, Dublin^37^24.8, Glasgow10	Not available
	HIV	–	0.5, Leicester^36^0.6, Birmingham^9^6.0, Dublin^37^	Not available
	Sexually transmitted diseases	1 (1.9)	9.4, Birmingham^9^8.0, Dublin 14	0.9–52.5, US^45^
Respiratory diseases3, (5.7)	COPD	1 (1.9)	1.7, Leicester^36^1.5, Birmingham^9^3.0, Dublin^37^	4–5, UK, Europe, and US^46^
	Asthma	1 (1.9)	16, Leicester^36^4.2, Birmingham^9^21, Dublin^37^	Not available
Endocrine disorders,1 (1.9)	Diabetes	1 (1.9)	2.8, Birmingham^9^8.0, Ireland^47^8.0, Dublin^37^7.3, Edinburgh^11^4.5, Glasgow^11^	8.0, US^41^6.1, Paris^39^4.0, Canada^38^
Gastrointestinal disorders,2 (3.8)	Gastrointestinal bleeds or ulcers	2 (3.8)	0.6, Birmingham^9^11.0, Dublin^37^14.7, Edinburgh^11^24.0, Glasgow^11^	Not available
Other,4 (7.5)	Leg ulcers	1 (1.9)	6.5, Birmingham^9^	Not available
	Learning disability	–	0.3, Birmingham^9^	36.0, Canada^48^
	Rheumatoid arthritis	–	0.1, Birmingham^9^6.0, Dublin^37^	Not available

^a^Blank fields may represent small numbers being suppressed for preserving patient anonymity. COPD = chronic obstructive pulmonary disease. HIV = human immunodeficiency virus.

### Multimorbidity

A total of 15/43 (34.9%) of PEH had two or more diagnosed health conditions. The age category with the greatest proportion of individuals suffering from multimorbidity (two or more diagnoses) was 50–59 years (85.7%).

### Prescribed medicines

The total number of prescriptions issued over the study period among the PEH registrants was 967. Over 95% of the patients (41/43) were prescribed at least one medicine, and 33/41 (80.5%) had been prescribed four or more concurrent medicines (Supplementary Table S1). Central nervous system (CNS) chapter of the *BNF* accounted for over a third of the prescriptions issued (36.1%). Of these prescriptions, 41.4% were for analgesic medications, of which the mean number of prescriptions issued per patient for both opioid and non-opioid analgesics was 3.4 (4.1 and 3.8, respectively). Additionally, 40.9% of the CNS-related prescriptions were associated with antidepressants, such as selective serotonin reuptake inhibitors (SSRIs), tricyclic antidepressants, and tetracyclic antidepressants. The second highest category for prescriptions was for infections, comprising 14.0% of the total number of prescriptions issued (Supplementary Table S1).

## Discussion

### Summary

This is the first study in the UK to investigate healthcare issues of PEH who utilise mainstream general practices. This study addresses the gap that exists in previous literature, which has largely explored the population registered with specialist homeless healthcare services.

The findings of this study show under-recording of homelessness in comparison with national homelessness rates, in an area expected to have higher rates owing to socioeconomic characteristics of the study region. In addition, the results also suggest a potential lack of utilisation of mainstream GP practices by PEH, thus accentuating their limited access to primary healthcare services. The 43 registrants who identified as PEH within this study accounted for 0.013% of the population within the region; a region that has four times as many statutory PEH compared with the English national average. It is clear that this observed proportion is several folds smaller in comparison with the PEH prevalence of 0.5% in the English general population.^2^

The low numbers of PEH identified through the data searches also suggests potential under-coding of homelessness in primary care medical records. When presenting to services, patients may not always declare their homelessness or may use their last permanent residence or temporary residence. The low numbers may also be explained by the itinerant nature of PEH, because patients may not reside for long enough at a fixed address within the mainstream practice’s local catchment area, leading to lack of opportunity for practices to fully register patients. As homelessness overlaps with mental ill health, substance misuse, and long-term health conditions, capitalising on windows of opportunity to register patients (for example, when presenting for help for acute conditions) is a starting point for consistent coding in medical records and a subsequent offer of tailored support.

### Strengths and limitations

This is the first study to investigate the healthcare issues of PEH in mainstream general practices. The electronic medical records software, EMIS, was used to collect the data for this study using a wide range of keywords for both homelessness and health conditions. This study did not investigate the forms of homelessness in the identified sample or the duration of their homelessness. Furthermore, data were unavailable to account for how often the registrants experiencing homelessness visited the mainstream general practices, analysis of which may have offered another useful indicator to help assess the extent of their utilisation of services. Although a total of 48 general practices were covered in the search of PEH, all belonged to one CCG, potentially limiting the generalisability of the findings.

### Comparison with existing literature

The demographic characteristics of the PEH in this study are comparable with the published data in England.^9^ However, the prevalence of key health conditions, particularly mental ill health and substance misuse, and multimorbidity, were far lower compared with the published literature, demonstrating potential underdiagnosis, undertreatment, or under-coding of key health conditions in this population. Previous literature has shown that despite their mean age being in the late thirties , the extent of multimorbidity is comparable with those in their late eighties .^10^ While these data suggest a need to improve screening of health conditions and their coding in mainstream GP practices, the way this is offered to patients with no fixed abode may influence the uptake and success of the service. For example, assertive outreach is likely to be more successful than static building-based service provision.

### Implications for practice and research

This study highlights the inequity of provision and access to healthcare services in primary care for PEH. Previous literature suggests that many PEH prefer to use specialist homeless healthcare services.^8^ While commissioning more specialist primary healthcare centres could be one way to improve access of health services to PEH, mainstream services need to be adapted to be inclusive of PEH. Previous studies show that frontline primary healthcare staff are often unaware of patient registration guidelines.^15^ There is a need for training and education of such staff to reinforce the registration guidelines allowing patients with no fixed abode to register without any problems. National distribution of ‘My right to access healthcare’ cards to PEH, as piloted in many geographical areas,^16^ should be extended. There is a need for health services to comply with the Homelessness Reduction Act 2017^17^ to ensure that hospitals refer and provide liaison for the patients to primary care services. In addition, education of healthcare professionals should incorporate the healthcare issues of PEH. Previous studies have also shown that perceived stigma and discrimination in mainstream general practices by healthcare professionals and other patients are key barriers to accessing services.^6^ Such factors also act as a barrier to patient transition from specialist homeless healthcare services to mainstream practices when patients find a permanent residence.^8^ Anti-stigma intervention for healthcare professionals can be useful.^18^

Appropriate coding of homelessness and associated health conditions is imperative to identify those in need of primary health care and to apply prevention measures. Recent studies conducted in primary care practices in Canada have demonstrated that screening patients for poverty, including housing insecurity, is feasible and lends to correct identification of patients facing social disadvantages.^19–21^ As inequalities such as poverty and homelessness are modifiable (similar to drug misuse or smoking), proactive screening of such social circumstances using validated tools, which are acceptable for both patients and healthcare professionals, can identify patients at risk and those already facing adversities, leading to appropriate coding and provision of tailored support. In addition, homelessness also appears as a code in the international classification of disease (ICD-11 — QD71), allowing clinicians to record such status where applicable.^22^ There is substantial literature showing the overlap between severe mental ill health, substance misuse, and early mortality.^23^ Appropriate coding will prompt signposting and referral to support programmes that are relevant to health conditions that overlap with homelessness, such as mental ill health and substance misuse. This will facilitate appropriate follow-up, screening, timely treatment, and management practices.

Low levels of primary healthcare access by PEH make them high users of emergency departments. However, they often present late to the service, and their mortality in the emergency department is shown to be 12 times higher than the general population.^24^ In addition to strengthening the inclusivity of mainstream practices to PEH, further research is also necessary to develop novel primary care services that can improve PEH access. Outreach-based interventions offered by non-medical prescribers, including pharmacists and nurses, have been shown to be effective in identifying undiagnosed health conditions and minimising use of emergency departments.^25,26^ Qualitative studies show that PEH value such dedicated services.^27,28^ Establishment of tailored interventions, including outreach-based services, have been advocated in the NHS *L* *ong* *T* *erm* *P* *lan*.^29^ There is also scope to widen the roles of community pharmacies as PEH utilise pharmacies on a regular basis for substance misuse treatment, needle exchange, and prescription collection.^30–33^ Clinical guidelines should be inclusive of social outcomes, such as homelessness, when providing services for substance misuse and severe mental illnesses.^34,35^ As this research only covered general practices within one CCG, a large-scale study capturing wider areas of the UK is needed to improve the generalisability of the findings. Larger national primary care databases, such as Clinical Practice Research Datalink and The Health Improvement Network database, can offer such opportunities. As the data in the present study compared health status of study participants with health status of PEH and general populations as reported in the published literature, future studies should consider a matched cohort design, with comparison of health status of PEH with the general populations within the same general practices.

In conclusion, this study demonstrates that PEH are under-represented in mainstream general practices. There is a need to improve access of PEH to mainstream care and improve coding of homelessness in patient medical records. As homelessness overlaps with mental ill health, substance misuse, and long-term health conditions, consistent coding of homelessness in medical records is imperative in order to offer tailored support when patients present for services.
